# Melatonin attenuates radiation-induced cortical bone-derived stem cells injury and enhances bone repair in postradiation femoral defect model

**DOI:** 10.1186/s40779-021-00355-y

**Published:** 2021-12-12

**Authors:** Wei Hu, Jia-Wu Liang, Song Liao, Zhi-Dong Zhao, Yu-Xing Wang, Xiao-Fei Mao, Si-Wei Hao, Yi-Fan Wang, Heng Zhu, Bin Guo

**Affiliations:** 1 Medical School of Chinese People’s Liberation Army (PLA), Beijing, 100853 China; 2 Department of Stomatology, the First Medical Centre, Chinese PLA General Hospital, Beijing, 100853 China; 3 Beijing Key Laboratory for Radiobiology, Beijing Institute of Radiation Medicine, Beijing, 100840 China

**Keywords:** Melatonin, Stem cells, Ionizing radiation, Bone regeneration

## Abstract

**Background:**

The healing of bone defects can be challenging for clinicians to manage, especially after exposure to ionizing radiation. In this regard, radiation therapy and accidental exposure to gamma (γ)-ray radiation have been shown to inhibit bone formation and increase the risk of fractures. Cortical bone-derived stem cells (CBSCs) are reportedly essential for osteogenic lineages, bone maintenance and repair. This study aimed to investigate the effects of melatonin on postradiation CBSCs and bone defect healing.

**Methods:**

CBSCs were extracted from C57BL/6 mice and were identified by flow cytometry. Then CBSCs were subjected to 6 Gy γ-ray radiation followed by treatment with various concentrations of melatonin. The effects of exogenous melatonin on the self-renewal and osteogenic capacity of postradiation CBSCs in vitro were analyzed. The underlying mechanisms involved in genomic stability, apoptosis and oxidative stress-related signaling were further analyzed by Western blotting, flow cytometry and immunofluorescence assays. Moreover, postradiation femoral defect models were established and treated with Matrigel and melatonin. The effects of melatonin on postradiation bone healing in vivo were evaluated by micro-CT and pathological analysis.

**Results:**

The decrease in radiation-induced self-renewal and osteogenic capacity were partially reversed in postradiation CBSCs treated with melatonin (*P* < 0.05). Melatonin maintained genomic stability, reduced postradiation CBSC apoptosis and intracellular oxidative stress, and enhanced expression of antioxidant-related enzymes (*P* < 0.05). Western blotting validated the anti-inflammatory effects of melatonin by downregulating interleukin-6 (IL-6) and tumor necrosis factor alpha (TNF-α) levels via the extracellular regulated kinase (ERK)/nuclear factor erythroid 2-related factor 2 (NRF2)/heme oxygenase-1 (HO-1) signaling pathway. Melatonin was also found to exhibit antioxidant effects via NRF2 signaling. In vivo experiments demonstrated that the newly formed bone in the melatonin plus Matrigel group had higher trabecular bone volume per tissue volume (BV/TV) and bone mineral density values with lower IL-6 and TNF-α levels than in the irradiation and the Matrigel groups (*P* < 0.05).

**Conclusion:**

This study suggested that melatonin could protect CBSCs against γ-ray radiation and assist in the healing of postradiation bone defects.

## Background

Gamma (γ)-rays are highly penetrating electromagnetic ionizing radiation that have been extensively applied in diagnostic radiography, radiation oncology, and military weapons [1]. Ionizing radiation has been reported to be closely associated with bone metabolism. Given its high calcium content, bone tissue is sensitive to ionizing radiation and absorbs nearly 40% more radiation than the surrounding tissues [2]. The cumulative incidence rate of pelvic fracture in women has been reported to be up to 13% after radiotherapy, while computer tomography imaging after stereotactic body radiotherapy in lung cancer patients frequently revealed rib fractures [3, 4]. Importantly, therapeutic or accidental ionizing radiation exposure could induce bone remodeling disorders, including malignancy, avascular necrosis, arrest of bone growth, fracture, and osteopenia [5, 6]. However, the mechanisms of radiation-induced bone loss at the cellular level are yet to be fully elucidated.

Cortical bone-derived stem cells (CBSCs) have been reported to possess increased clonal incidence, potency, and developmental capacity compared to their bone marrow-derived counterparts [7]. Due to their superior proliferative and differentiation abilities, CBSCs may be an alternative approach for regenerative medicine [8]. It is of considerable interest to design a safe and effective treatment approach for postradiation bone damage, emphasizing the need to search for novel pharmacological interventions to treat postradiation bone damage. Collectively, radioprotective agents are conventionally divided into five categories: sulfydryl compounds, cytokines, hormones, antioxidants, and traditional Chinese medicine [9–11].

In human, the pineal gland primarily synthesizes and secretes the hormone N-acetyl-5-methoxytryptamine (melatonin). Melatonin acts as an autocrine, paracrine and endocrine hormone that regulates the circadian rhythms of neighboring and distant cells [12]. Furthermore, melatonin is a potent free radical scavenger that efficiently removes singlet oxygen, superoxide anion radicals, and hydroperoxide and importantly enhances the expression of antioxidative enzymes, such as superoxide dismutase, glutathione peroxidase, and catalase [13]. A systematic review by Zetner et al. [14] reported that exogenous melatonin reduced oxidative stress and inflammation in all studied animal tissues and improved 30-day survival. Additionally, melatonin reportedly modulates immune responses and exhibits anti-aging properties. In this respect, Serin et al. showed that administration of 100 mg/kg of melatonin to the lungs of rats before γ-ray exposure reduced alveolar edema, macrophage and lymphocyte infiltration [15]. Notably, melatonin could stimulate osteogenic and chondrogenic differentiation but inhibit adipogenic differentiation of bone marrow-derived mesenchymal stem cells (BMMSCs) [16]. Dong et al. [17] demonstrated that melatonin drove the differentiation of mesenchymal stem cells into osteogenic lineages via neuropeptide Y signaling. Nonetheless, little is known about the therapeutic effects of melatonin in radiation-induced bone changes.

In this study, we hypothesized that melatonin would alleviate radiation-induced oxidative stress and restore the osteogenic capacity of postradiation CBSCs. We further explored whether melatonin was helpful in the healing of postradiation bone defects and investigated the underlying molecular mechanisms.

## Methods

### Animals

C57BL/6 mice aged eight weeks were procured from Vital River Experimental Animal Technology Co., Ltd. (Beijing, China). All animal experiments were approved by the Animal Ethics Committee of the Academy of Military Medical Sciences. All surgical and irradiation procedures were performed under anesthesia, and animal suffering was minimized as much as possible.

### Isolation, culture, and identification of CBSCs

CBSCs were isolated as previously described [18]. Briefly, the femurs were extracted after mice execution. Sterile scissors were used to remove the epiphyses just below the end of the marrow cavity, followed by thorough washing of the bone cavities using alpha minimal essential medium (α-MEM) until the bones appeared pale. The excised compact bones were excised into chips of approximately 3 mm^3^, and were transferred into Eppendorf tubes containing 1 ml of α-MEM and 1 mg/ml of collagenase II at 37 °C for 1 h, followed by incubation with normal growth medium. Peridinin chlorophyll protein complex (PerCP)-conjugated anti-mouse CD45 antibodies and Phycoerythrin (PE)-conjugated anti-mouse spinocerebellar ataxia type-1 (Sca-1), CD140a, CD105, CD80, CD44, CD31, and CD11b antibodies (eBioscience, Cambridge, UK) were used to stain CBSCs in the dark according to the manufacturer’s instructions. Then flow cytometry analysis was performed using a FACSCalibur flow cytometer (BD Biosciences, San Jose, CA, USA) and data were analyzed using the FlowJo V10 software (BD Biosciences).

### Irradiation

A Cobalt-60 radiation facility at the Institute of Radiation Medicine, the Academy of Military Medical Sciences (Beijing, China), was used for irradiation [19]. Six Gy γ-rays at dose rates of 0.5–1 Gy/min were applied to cells and mice, with the field uniformed within ± 2%. All the steps for irradiation were performed by specialists of Beijing Key Laboratory for Radiobiology (China). Except the hind legs and lower abdomens, the whole mouse body was obstructed and protected from γ-rays using lead blocks.

### Experimental design and tests

One hour later after radiation, CBSCs were treated with various concentrations of melatonin for 24 h. Then, the melatonin-containing medium was replaced by a normal growth medium. Cells were divided into five groups: control, irradiation (IR), irradiation plus 1 μmol/L melatonin treatment (IR + LMLT), irradiation plus 10 μmol/L melatonin treatment (IR + MMLT), irradiation plus 100 μmol/L melatonin treatment (IR + HMLT). Cells from passages 3 to 5 were used for subsequent in vitro experiments.

#### Analysis of colony-forming unit fibroblast (CFU-F)

CBSCs were seeded into 6-well plates at a density of 200 cells/well and cultured for 12 days. When visible colonies were formed, the colonies were fixed with 20% methanol, stained with 0.1% crystal violet, and photographed. Finally, positive colony formation (more than 50 cells/colony) was determined by counting under a microscope.

#### Osteogenesis and adipogenesis assay

The culture media for differentiation were purchased from Cyagen Biosciences (Santa Clara, CA, USA). Total RNA was extracted for quantitative real-time reverse transcription-polymerase chain reaction (qRT-PCR) after osteogenic and adipogenic differentiation. Alkaline phosphatase staining was performed according to the manufacturer’s instructions (Beyotime) after two weeks of osteogenic differentiation. Alizarin red S staining was performed to assess calcium deposition after three weeks of osteogenic differentiation. Lipid droplets were visualized using filtered working Oil red O solution after two weeks of adipogenic differentiation.

#### Cell apoptosis analysis

An Annexin V-Allophycocyanin (APC)/7-Aminoactinomycin D (7-AAD) apoptosis detection kit (Keygen, Jiangsu, China) was used to determine apoptosis of CBSCs. Briefly, the cells were re-suspended in 500 µl of binding buffer. Then, 5 µl of APC and 5 µl of 7-AAD were added to the suspension and incubated for 15 min at room temperature in the dark. Cell apoptosis was determined by flow cytometry for 2 × 10^4^ events at a flow rate not exceeding 500 cells per second, and the data were analyzed using the FlowJo V10 software.

#### Determination of ROS, MDA, SOD levels and GSH/GSSG ratio

Intracellular reactive oxygen species (ROS) levels were investigated using a ROS assay kit (Beyotime). Briefly, the medium was replaced with a serum-free medium containing a 2′,7′-dichlorodihydrofluorescein diacetate (DCFH-DA) probe at a final concentration of 10 µmol/L and placed in the dark for 30 min. The cells were then harvested, and fluorescence detection was performed using flow cytometry at an emission wavelength of 525 nm and an excitation wavelength of 488 nm. The measurements of intracellular malondialdehyde (MDA), superoxide dismutase (SOD) levels and the glutathione/oxidized glutathione (GSH/GSSG) ratio were performed using an MDA assay kit (Ray Biotech), a SOD assay kit (Beyotime) and a GSH/GSSG assay kit (Beyotime) according to the manufacturers’ instructions.

#### qRT-PCR

The TRIzol reagent (Invitrogen, Carlsbad, CA, USA) was used to isolate total RNA from the cells. A reverse transcription kit (Yishan, Shanghai, China) was then used to reverse transcribe the RNA to cDNA. The quantitative real-time PCR reaction was performed according to the reagent instructions of the UltraSYBR Mixture kit (CWBIO, Beijing, China), using the following reaction program: 95 °C for 2 min, followed by 40 cycles of 95 °C for 15 s, 60 °C for 30 s and 72 °C for 30 s. Sangon Biotech (Shanghai, China) synthesized all the primers. The primers used in this study are listed as follows (5′-3′): *Runx2*, sense, GACTGTGGTTACCGTCATGGC and anti-sense, ACTTGGTTTTTCATAACAGCGGA; *Opn*, sense, ATCTCACCATTCGGATGAGTCT and antisense, TGTAGGGACGATTGGAGTGAAA; *Pparg*, sense, GGAAGACCACTCGCATTCCTT and antisense, GTAATCAGCAACCATTGGGTCA; *Cebpa*, sense, GCGGGAACGCAACAACATC and antisense, GTCACTGGTCAACTCCAGCAC. Relative gene expression was quantified using the 2^−ΔΔCt^ method [20].

#### Immunofluorescence

Cells were seeded into 12-well plates plated with cell-climbing slices at a 2 × 10^4^/well density. Paraformaldehyde was then used to fix the cells, and 0.5% Triton X-100 was used to permeabilize them. Goat serum was then used to block non-specific binding to the cells before incubation with antibodies against gamma H2A histone family member X (γH2AX; 1:200; CST, Danvers, MA, USA) overnight. The cells were then incubated with secondary antibodies conjugated to a fluorescent dye. Nuclear counterstaining was achieved using 4′,6-diamidino-2-phenylindole (DAPI) at room temperature. The coverslips with the cells were inverted on glass slides, and fluorescence was assessed under a laser confocal microscope.

#### Western blotting analysis

Proteins were extracted as previously described [19]. Subsequently, SDS-PAGE (7.5–10% polyacrylamide gels) was used to separate the proteins, which were then transferred to polyvinylidene fluoride (PVDF) membranes (Thermo Scientific). The membranes were blocked with 5% skim milk and then incubated with the primary antibodies (1:1000; CST, Danvers, MA, USA) overnight, followed by incubation with secondary antibodies (1:2000; CST, Danvers, MA, USA) at room temperature. The immunoreactive protein bands were visualized using an enhanced chemiluminescence (ECL) kit (Thermo Scientific). A Luminescent Image Analyzer LAS4000 (Fuji Film, Tokyo, Japan) and ImageJ software (NIH, Bethesda, MD, USA) were used to detect and quantify the protein band signals. U0126 ERK pathway inhibitor was obtained from Sigma-Aldrich (Danvers, MA, USA). ML385 NRF2 pathway inhibitor was purchased from MedChemExpress (Shanghai, China).

### Femoral bone defect models

One hour later after radiation, the mice were subject to bone defect surgery. A femoral defect (1.5 mm diameter and 1 mm depth) was generated at the distal third of the femur, as previously described [19]. Forty mice were randomly and equally divided into four groups: control (*n* = 10), irradiation (IR, *n* = 10), irradiation plus Matrigel treatment (IR + gel, *n* = 10), irradiation plus Matrigel with 100 μmol/L melatonin treatment (IR + gel + MLT, *n* = 10). Surgically treated, nonirradiated mice were used as blank controls. Melatonin was first diluted in absolute ethanol and then mixed and diluted with Matrigel at 4 °C to achieve a concentration of 100 µmol/L. Matrigel is liquid at 4 °C and forms a semi-solid gel at 37 °C. A 10 µl aliquot of Matrigel mixed with melatonin was injected into the postradiation femoral defect. The same volume of Matrigel without melatonin was used as a control. The mice were sacrificed four weeks after surgery, and micro-CT analysis and pathological evaluation of the harvested femurs were performed.

#### Micro-CT scan

The femur samples were fixed in formaldehyde and then placed in a 50 mm diameter tube oriented perpendicular to the scanning axis. Bone samples were then scanned using a Quantum GX micro-CT imaging system (PerkinElmer, Waltham, MA, USA) using the following settings: 70 kV, 100 µA and 14 min exposure time. A selected area (27 mm^3^) of the three-dimensional reconstruction was centered at bone drilling site, followed by determination of the bone mineral density (BMD) and trabecular bone volume per tissue volume (BV/TV).

#### Hematoxylin–eosin staining, Masson staining and immunohistochemistry

Femurs were obtained, fixed in formaldehyde, decalcified, and embedded in paraffin. Femur sections were deparaffinized in xylene, dehydrated in ethanol and rinsed with tap water. Then, these sections were stained with hematoxylin and eosin solution (H&E staining) each for 5 min or Lichun red magenta and aniline blue solution (Masson staining) each for 5 min. To prepare for immunohistochemistry (IHC) analysis, the tissue sections were subjected to the same dehydration protocol before antigen retrieval. Tumor necrosis factor alpha (TNF-α) and interleukin-6 (IL-6) primary antibodies (1:100) were added to the tissues and incubated overnight. Subsequently, these sections were incubated with secondary antibodies labeled with horseradish peroxidase. Diaminobenzidine was added to these tissues for color development. The tissue sections were then counterstained with hematoxylin, dehydrated, incubated with xylene to make them transparent. All tissue sections were sealed with neutral balsam followed by observation under an optical microscope.

### Statistical analysis

The mean ± standard deviation from at least three independent experiments was used to represent all the quantitative variables in the present study. GraphPad Prism 6.02 (GraphPad software Inc., San Diego, CA, USA) was used to conduct the statistical analyses. One-way analysis of variance was used to compare the data from multiple groups. A *P* value less than 0.05 was statistically significant.

## Results

### Melatonin attenuates radiation-induced CBSCs injury

#### Melatonin alleviates radiation-induced the loss of self-renewal and osteogenic capacity of CBSCs

**Fig. 1:**
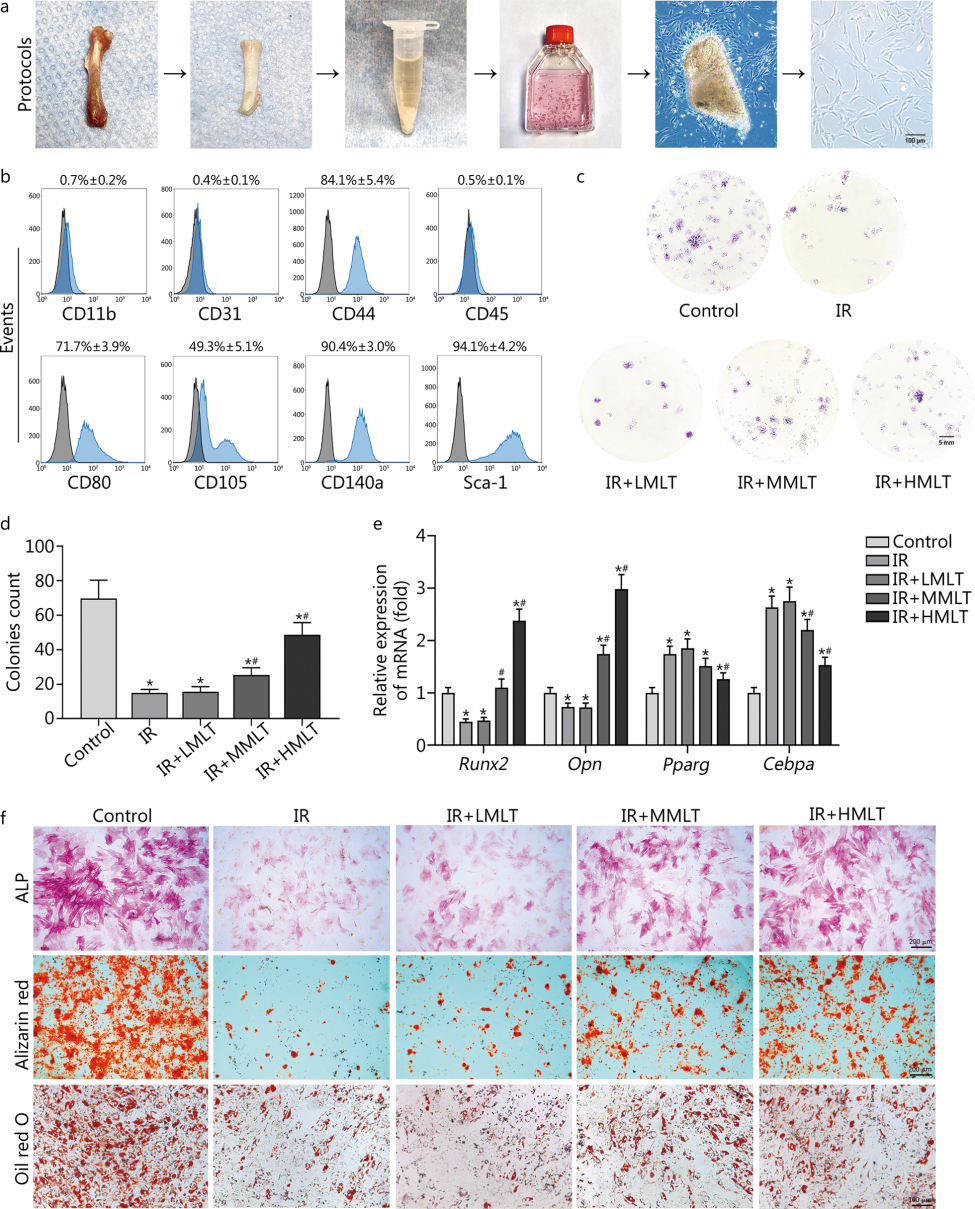
Effect of melatonin on self-renewal and multi-directional differentiation potential of postradiaiton CBSCs. **a** Protocols for extracting CBSCs. **b** Identification of CBSCs by flow cytometry. **c**, **d** Colonies count for detecting self-renewal ability. **e** qRT-PCR analysis of osteogenic and adipogenic gene markers. **f** ALP staining and Alizarin red staining after osteogenic induction. Oil red O staining after adipogenic induction. Compared with control, ^*^P < 0.05; compared with IR, ^#^*P* < 0.05. IR irradiation, IR + LMLT irradiation plus low 1 μmol/L melatonin treatment, IR + MMLT irradiation plus medium 10 μmol/L melatonin treatment, IR + HMLT irradiation plus high 100 μmol/L melatonin treatment, CBSCs cortical bone-derived stem cells, ALP alkaline phosphatase

We extracted and cultured CBSCs from femurs according to previously described protocols (Fig. 1a). The results for CBSCs characterization showed that CBSCs were positive for CD44, CD80, CD105, CD140a and Sca-1 markers, but negative for CD11b, CD31 and CD45 markers (Fig. 1b). To assess whether melatonin could affect radiation-induced injury, CBSCs received a single dose of 6 Gy γ-ray radiation with various melatonin concentrations. Radiation-induced impairment of the self-renewal ability of CBSCs was demonstrated, and 100 μmol/L melatonin was found to significantly increase colony formation compared to the IR group (*P* < 0.05) (Fig. 1c, d). Furthermore, higher expression levels of osteogenic gene markers *Runx2* and *Opn* were observed after treatment with 100 μmol/L melatonin, compared to the IR group (*P* < 0.05). Conversely, radiation promoted the adipogenic differentiation of CBSCs, and 100 μmol/L melatonin significantly decreased the expression of adipogenesis-associated genes *Pparg* and *Cebpa* (*P* < 0.05) (Fig. 1e). In addition, ALP is an early osteogenic differentiation marker, and calcium deposition determined by Alizarin red staining was used as a late osteogenic differentiation marker. ALP staining and Alizarin red staining results were consistent with the expression trend of osteogenic genes. However, lipid droplets representing adipogenic activity were found fewer in the IR group than in control and 100 μmol/L melatonin treatment groups, which was likely related to changes in cell viability (Fig. 1f).

#### Melatonin maintains the genomic stability and attenuates apoptosis of postradiation CBSCs

**Fig. 2:**
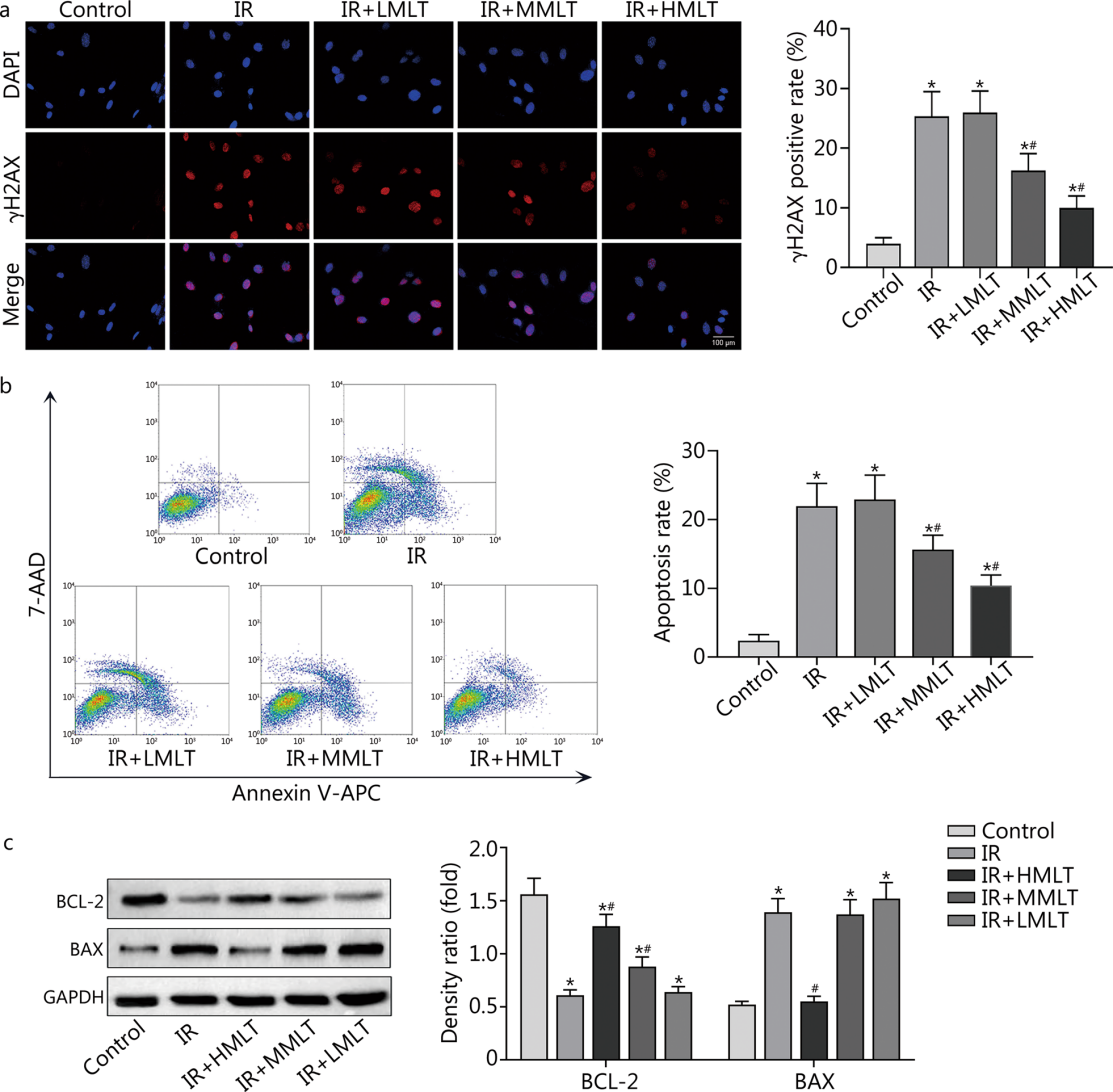
Effect of melatonin on DNA damage and apoptosis of postradiation CBSCs. **a** Analysis of DNA breaks using γH2AX immunostaining. DAPI (blue) and γH2AX (red). **b** Analysis of apoptosis by flow cytometry. **c** Levels of anti-apoptotic protein BCL-2 and pro-apoptotic protein BAX were measured by Western blotting. Compared with control, ^*^P < 0.05; compared with IR, ^#^*P* < 0.05. IR irradiation, IR + LMLT irradiation plus low 1 μmol/L melatonin treatment, IR + MMLT irradiation plus medium 10 μmol/L melatonin treatment, IR + HMLT irradiation plus high 100 μmol/L melatonin treatment, CBSCs cortical bone-derived stem cells, γH2AX gamma H2A histone family member X, BCL-2 B-cell lymphoma-2, BAX BCL2-associated X

γH2AX has been reported to be a biomarker for DNA double-strand breaks (DSBs). In the present study, fluorescence-labeled γH2AX was found in the nuclei of CBSCs. No significant difference was found between IR and 1 μmol/L melatonin treatment groups; however, a significant decrease was observed in other melatonin treatment groups (*P* < 0.05) (Fig. 2a). Previous studies have shown that DSBs could induce apoptosis. Flow cytometry results showed that treatment with 10 μmol/L or 100 μmol/L melatonin significantly attenuated apoptosis of postradiation CBSCs (*P* < 0.05) (Fig. 2b). Furthermore, a significant increase in anti-apoptotic protein BCL-2 was observed with 10 μmol/L and 100 μmol/L melatonin, which was the opposite of changes observed for pro-apoptotic protein BAX (*P* < 0.05) (Fig. 2c).

#### Melatonin inhibits intracellular oxidative stress and enhances antioxidant enzymes activity in postradiation CBSCs

**Fig. 3:**
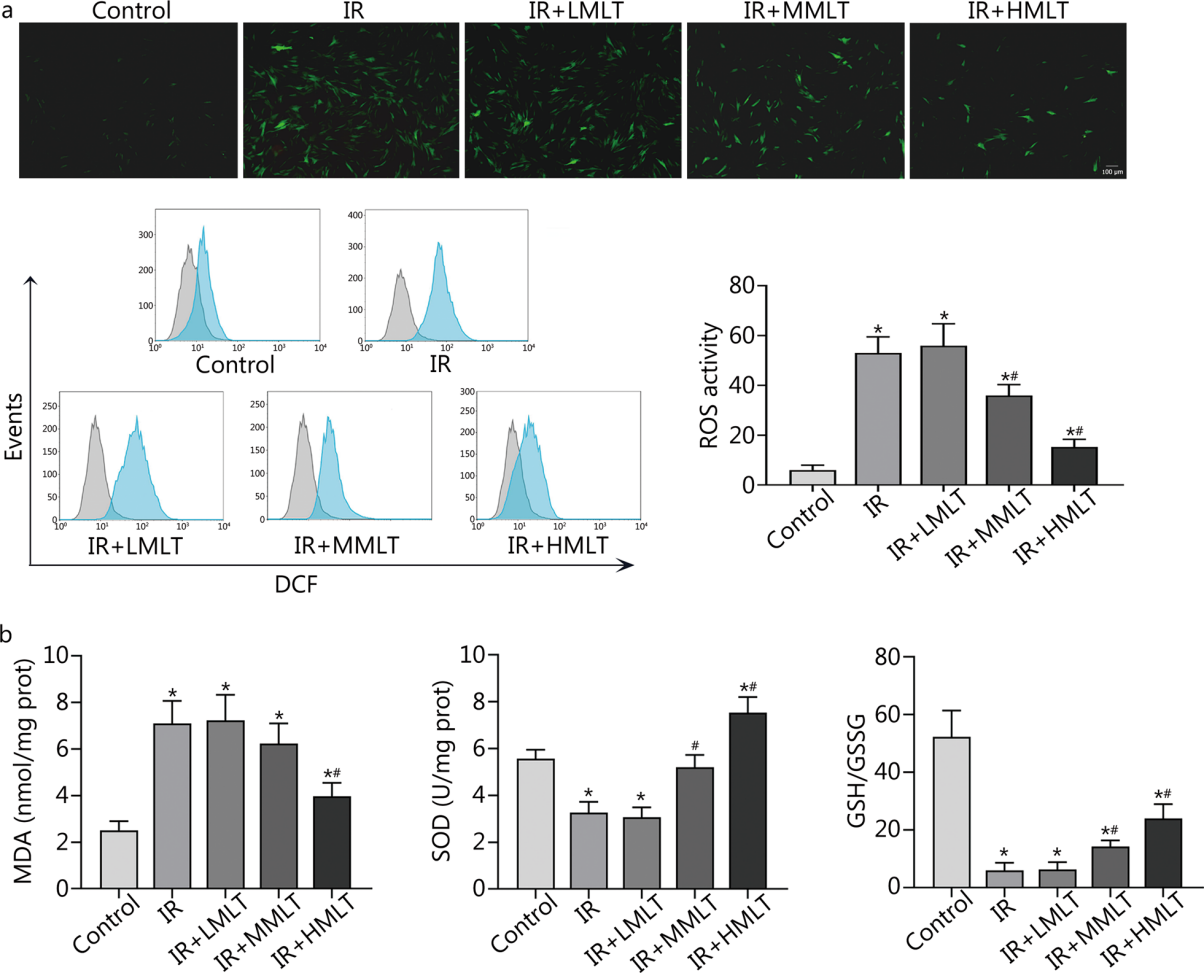
Effect of melatonin on intracellular oxidative stress in postradiation CBSCs. **a** Intracellular ROS levels were detected and quantified by flow cytometry after DCFH-DA staining. **b** Levels of oxidative stress-related markers in CBSCs. Compared with control, ^*^P < 0.05; compared with IR, ^#^*P* < 0.05. IR irradiation, IR + LMLT irradiation plus low 1 μmol/L melatonin treatment, IR + MMLT irradiation plus medium 10 μmol/L melatonin treatment, IR + HMLT irradiation plus high 100 μmol/L melatonin treatment, CBSCs cortical bone-derived stem cells, ROS reactive oxygen species, DCFH-DA 2,7-dichlorodihydrofluorescein diacetate, DCF 2',7'-dichlorofluorescein, MDA malondialdehyde, SOD superoxide dismutase, GSH/GSSG glutathione/oxidized glutathione

Quantification of intracellular reactive oxygen species (ROS) levels was performed by 2′,7′-dichlorodihydrofluorescein diacetate (DCFH-DA) staining. DCFH-DA was hydrolyzed and oxidized to generate 2′,7′-dichlorofluorescein (DCF) with green fluorescence. As shown in Fig. 3a, postradiation CBSCs with or without 1 μmol/L melatonin treatment exhibited brighter green fluorescence than the other groups. We further conducted a quantitative analysis of ROS by flow cytometry and found that irradiation triggered a surge in ROS levels, while 100 μmol/L melatonin treatment remarkably decreased ROS production in postradiation CBSCs (*P* < 0.05) (Fig. 3a). As seen in Fig. 3b, the melatonin-treated postradiation CBSCs had lower MDA content, higher levels of SOD and higher GSH/GSSG ratios compared to the IR group (*P* < 0.05). It is widely acknowledged that GSH exists in reduced and GSSG, and GSH can be oxidized into GSSG by ROS. Given that treatment with 100 μmol/L melatonin yielded optimal results in the healing of postradiation CBSCs, the mechanisms underlying the protective effects of melatonin were further investigated.

#### Melatonin exerts anti-inflammatory effect via the ERK/NRF2/HO-1 signaling and exerts antioxidant effect via NRF2 signaling

**Fig. 4:**
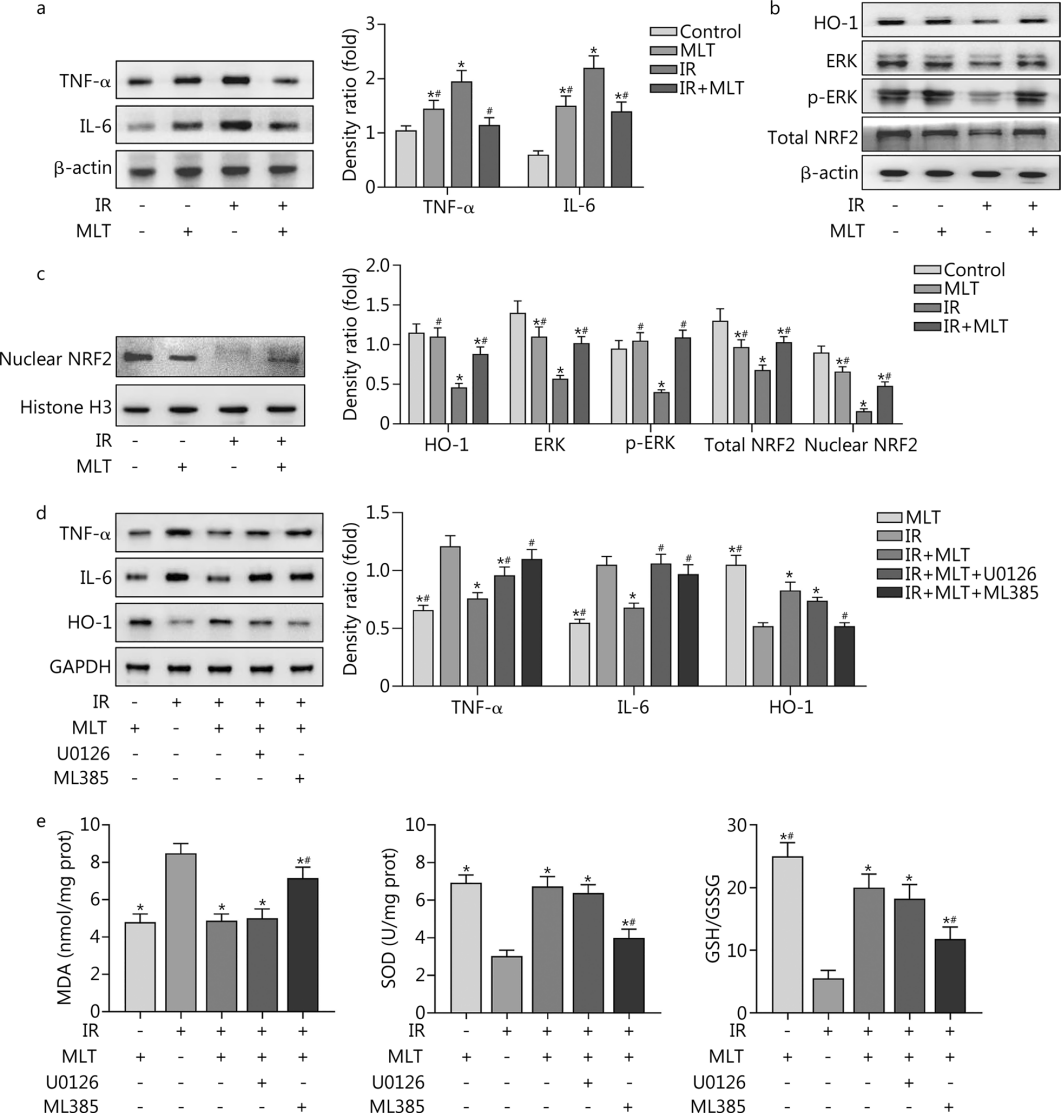
Melatonin activates ERK/NRF2/HO-1 signaling pathway in postradiation CBSCs. **a** Levels of TNF-α and IL-6 in CBSCs were detected by Western blotting. **b**, **c** Intracellular markers of ERK/NRF2/HO-1 signaling were measured by Western blotting. ^*^P < 0.05 compared with control; ^#^*P* < 0.05 compared with IR. **d** Levels of HO-1, TNF-α and IL-6 were detected after postradiation CBSCs were treated with melatonin and inhibitors. **e** Levels of oxidative stress-related markers were measured after postradiation CBSCs were treated with melatonin and inhibitors. Compared with IR, ^*^P < 0.05; compared with IR + MLT, ^#^*P* < 0.05. IR irradiation, MLT melatonin, CBSCs cortical bone-derived stem cells, ERK extracellular regulated kinase, NRF2 nuclear factor erythroid 2-related factor 2, HO-1 heme oxygenase-1, TNF-α tumor necrosis factor alpha, IL-6 interleukin-6

As shown in Fig. 4a, irradiation resulted in a significant increase in TNF-α and IL-6 levels, whereas 100 μmol/L melatonin significantly reversed these alterations (*P* < 0.05). To determine whether the extracellular regulated kinase (ERK)/nuclear factor erythroid 2-related factor 2 (NRF2)/heme oxygenase-1 (HO-1) signaling pathway played a role in the anti-inflammatory and antioxidant effects of melatonin, the nucleoproteins and total proteins of CBSCs were extracted for Western blotting analysis. As seen in Fig. 4b, c, the protein levels of ERK, phosphorylated (p)-ERK, total NRF2, HO-1 and nuclear NRF2 were significantly repressed in response to radiation, while melatonin significantly activated those signaling markers respectively, compared to the IR group (*P* < 0.05). Slight differences in ERK and NRF2 protein levels induced by melatonin toxicity were observed between the control and MLT groups (*P* < 0.05). To further validate the involvement of the ERK/NRF2/HO-1 pathway in the regulation of downstream molecules, we utilized U0126 (an ERK inhibitor) and ML385 (an NRF2 inhibitor) to suppress the pathway. As seen in Fig. 4d, the inhibition of TNF-α and IL-6 was reversed by U0126 and ML385 inhibitors (*P* < 0.05), suggesting the anti-inflammatory effects of melatonin. As seen in Fig. 4e, the alterations of MDA, SOD and GSH/GSSG were reversed by the ML385 inhibitor instead of the U0126 inhibitor (*P* < 0.05), indicating the antioxidant effect of melatonin. These results indicated that melatonin exerted its anti-inflammatory effects via activation of the ERK/NRF2/HO-1 signaling pathway and exerted its antioxidant effects via activation of the NRF2 signaling pathway.

### Melatonin promotes bone healing of postradiation femoral defect in vivo

**Fig. 5:**
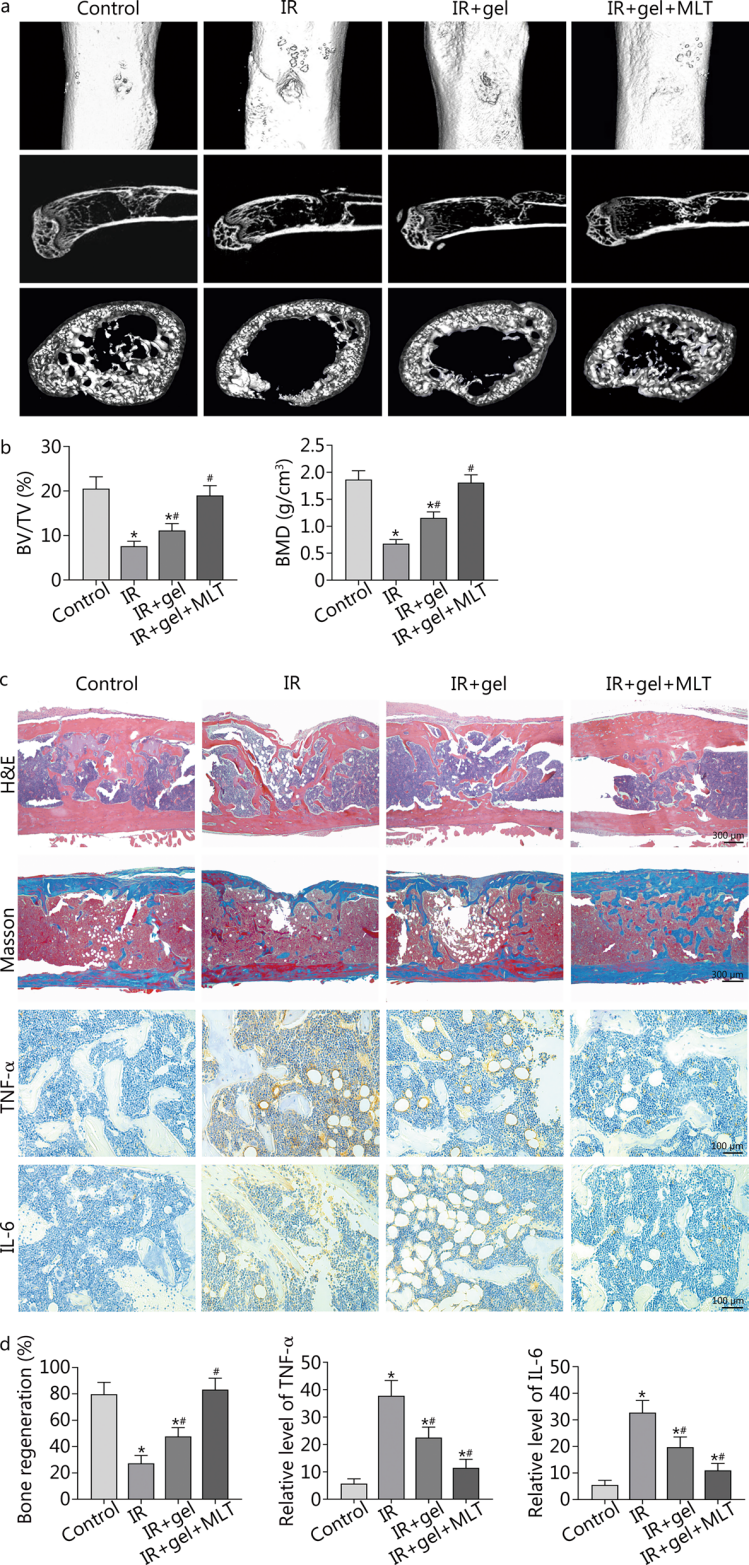
Melatonin benefits bone healing in a postradiation femoral defect model. **a**, **b** Representative three-dimensional images and quantitative analysis of micro-CT. **c**, **d** Representative images of H&E staining, Masson staining and immunochemical staining. Quantitative analysis was performed. Compared with control, ^*^P < 0.05; compared with IR, ^#^*P* < 0.05. IR irradiation, gel Matrigel, MLT melatonin, BV/TV bone volume/tissue volume, BMD bone mineral density, H&E hematoxylin–eosin, TNF-α tumor necrosis factor alpha, IL-6 interleukin-6

A femoral bone defect model was established to investigate the potential role of melatonin in bone repair in vivo, as previously described. Given its abundant extracellular matrix, BD Matrigel was chosen to be a melatonin carrier that transformed from a liquid state to a semi-solid gel from 4 to 37 °C. Continuous osteogenesis that bridged the cortical defects was observed in the control and the melatonin treatment groups. Quantitative results revealed that melatonin treatment significantly improved bone parameters, including BV/TV and BMD values, compared to the IR and Matrigel groups (*P* < 0.05) (Fig. 5a, b). To substantiate the imaging results, representative histological and immunohistochemical sections of the healed bone defects were assessed. H&E and Masson staining results were consistent with micro-CT imaging findings indicating that melatonin enhanced bone regeneration in the postradiation defect regions. Moreover, protein levels of pro-inflammatory cytokines TNF-α and IL-6 in the defect regions were significantly increased after radiation and were significantly decreased after melatonin treatment (*P* < 0.05) (Fig. 5c, d).

## Discussion

It is widely acknowledged that exposure to radiation can result from military nuclear weapons, nuclear accidents, and radiotherapy. However, little is known on the plethora of complications elicited, including prolonged healing of bone trauma. The adverse effects of high-dose radiation on bone, including sustained hypoxia of small blood vessels, decreased activity and abundance of osteocytes and osteoblasts, reportedly lead to cytopenia and even radionecrosis [21, 22]. Herein, we delivered a single sublethal dose of 6 Gy γ-ray radiation to CBSCs and established a mouse model of postradiation bone defect. Importantly, instead of using allogeneic stem cells delivery, the combination of melatonin with Matrigel used yielded a good performance in maintaining the self-renewal and osteogenic capacity of CBSCs in situ*.*

Interestingly, stem or progenitor cells have been documented to be more sensitive to radiation than non-proliferating and highly differentiated cells [23]. In the present study, it was difficult to ascertain whether stem cells from the cortical bone or the bone marrow played a critical role in healing the femoral defects. The concept of skeletal stem cells has recently been proposed, and various markers of differentiated subgroups of skeletal stem cells residing in the growth plate, periosteum, have already been identified [24–26]. BMMSCs consist of a heterogeneous cell mixture, and only a fraction of these cells possesses stem cell properties. Previous studies suggested that the cortical bone, rather than the bone marrow, might be a source of stem cells for regeneration, given its more primitive characteristics [27]. Moreover, the therapeutic properties of CBSCs have been applied for cardiac wound healing [28].

Melatonin is well known for its potent antioxidant capacity, low toxicity, and wide distribution throughout the body [29]. Melatonin and its metabolites can efficiently neutralize free radicals. Notably, melatonin is an amphiphilic peptide and has receptor-dependent and -independent functions [30]. This evolutionarily conserved hormone achieves multiple regulatory functions via binding to specific high-affinity receptors, namely MT1/MT2, on the plasma membrane. Melatonin receptors MT1 and MT2 have been reported to be expressed in BMMSCs [31]. Melatonin-mediated osteogenic promotion has been reported to be dependent on the presence of MT2 receptors in BMMSCs [32]. He et al. [33] found that melatonin resulted in a superior ability to scavenge free radicals, enhance proliferation and osteogenic capacity, and downregulate the expression of matrix metalloproteinase in BMMSCs under the synergistic effect of extracellular matrix (ECM). Based on these findings, we speculated that Matrigel, which is rich in ECM and provides cells with the environment to form three-dimensional structures, combined with melatonin, could benefit postradiation bone healing.

To date, many studies have investigated the molecular mechanisms underlying the immunoregulatory, antioxidative, and bone-supportive properties of melatonin. Pharmacological doses of melatonin were found to inhibit IFN-γ production in the range of 0.1 mmol/L to 1 mmol/L [34]. Oxidative stress and inflammation are interconnected pathophysiological processes associated with various inflammatory diseases [35]. In the present study, we demonstrated that exogenous melatonin inhibited ROS production and attenuated the inflammatory response in postradiation bone. Oxidative stress is thought to contribute significantly to DNA damage, apoptosis and senescence. Mechanistically, excessive oxidative stress can upregulate the tumor suppressors p53 and pRB. Activated p53 results in cell cycle arrest by transcriptional regulation of p21, leading to apoptosis [36, 37]. On the other hand, inflammatory cytokines are important for the stemness and differentiation of BMMSCs. TNF-α and IL-6 have been shown to promote apoptosis through NO regulation and inhibit the expression of transcription factors Osterix and Runx2 [38]. Interestingly, Wang et al. [39] found that melatonin could reverse the loss of stemness induced by TNF-α in human BMMSCs through activation of YAP expression. In addition, NRF2 signaling acts as a master regulator of antioxidative stress, regulating antioxidant responsive gene expression and phase II detoxifying enzymes such as NQO1 and HO-1, which remove cytotoxic ROS to counteract oxidative damage [40]. Studies have shown that wogonin exerted an anti-inflammatory effect through the ROS/ERK/NRF2 signaling pathway [41], and NRF2 deficiency enhanced inflammatory disorder susceptibility [42]. Likewise, our results indicated that the ERK/NRF2/HO-1 signaling pathway activated by melatonin suppressed the inflammatory responses. However, unlike the NRF2 inhibitor, the ERK inhibitor failed to block the alleviating effects of melatonin in the present study. Further studies on other upstream regulatory molecules of NRF2, and the lipophilic properties of melatonin could provide more explanation for this phenomenon.

Nonetheless, several limitations should be further addressed. The effect of different radiation doses, the timing of melatonin treatment intervention and associated outcomes should be further explored. Whether the receptor-dependent function of melatonin is involved in inflammation and oxidative stress in postradiation CBSCs should be thoroughly investigated from a mechanical aspect. Systemic use of melatonin could be a more reasonable approach to manage total body irradiation-induced bone injury.

## Conclusion

The therapeutic use of melatonin could restore the self-renewal and osteogenic capacity of postradiation CBSCs by maintaining genomic stability and reducing apoptosis. Melatonin exerted anti-inflammatory effects via the ERK/NRF2/HO-1 signaling pathway and antioxidant effects via NRF2 signaling. Further analysis revealed that melatonin promoted the repair of postradiation bone defects and downregulated TNF-α and IL-6 levels in bone defect regions. Overall, our results suggest that melatonin could exert clinical benefits in the treatment of postradiation bone defects.

## Data Availability

Not applicable.

## Abbreviations

ALP: Alkaline phosphatase
α-MEM: Alpha minimal essential medium
APC: Allophycocyanin
BAX: BCL2-associated X
BCL-2: B-cell lymphoma-2
BMD: Bone mineral density
BMMSCs: Bone marrow-derived mesenchymal stem cells
BV/TV: Bone volume/tissue volume
CBSCs: Cortical bone-derived stem cells
CFU-F: Colony-forming unit fibroblast
DCF: 2′,7′-Dichlorofluorescein
DCFH-DA: 2′,7′-Dichlorodihydrofluorescein diacetate
DSBs: DNA double-strand breaks
ECM: Extracellular matrix
ERK: Extracellular regulated kinase
gel: Matrigel
GSH/GSSG: Glutathione/oxidized glutathione
H&E: Hematoxylin and eosin
HO-1: Heme oxygenase-1
IHC: Immunohistochemistry
IL-6: Interleukin-6
IR: Irradiation
MDA: Malondialdehyde
MLT: Melatonin
NRF2: Nuclear factor erythroid 2-related factor 2
PerCP: Peridinin chlorophyll protein complex
qRT-PCR: Quantitative real-time reverse transcription-polymerase chain reaction
ROS: Reactive oxygen species
Sca-1: Spinocerebellar ataxia type-1
SOD: Superoxide dismutase
TNF-α: Tumor necrosis factor alpha
γH2AX: Gamma H2A histone family member X
7-AAD: 7-Aminoactinomycin D
